# The clinical course and pathophysiological investigation of adolescent gestational diabetes insipidus: a case report

**DOI:** 10.1186/s12902-018-0234-6

**Published:** 2018-01-30

**Authors:** Tatsuya Kondo, Miwa Nakamura, Sayaka Kitano, Junji Kawashima, Takeshi Matsumura, Takashi Ohba, Munekage Yamaguchi, Hidetaka Katabuchi, Eiichi Araki

**Affiliations:** 1 Department of Metabolic Medicine, Faculty of Life Sciences, Kumamoto University, 1-1-1 Chuo-Ward, Honjo, Kumamoto, 860-8556 Japan; 2 Department of Obstetrics and Gynecology, Faculty of Life Sciences, Kumamoto University, 1-1-1 Chuo-Ward, Honjo, Kumamoto, 860-8556 Japan

**Keywords:** Gestational diabetes insipidus, Vasopressinase (=insulin regulated aminopeptidase: IRAP), Glucose transporter 4

## Abstract

**Background:**

Gestational diabetes insipidus (GDI) is a rare endocrine complication during pregnancy that is associated with vasopressinase overproduction from the placenta. Although increased vasopressinase is associated with placental volume, the regulation of placental growth in the later stage of pregnancy is not well known.

**Case presentation:**

A 16-year-old pregnant woman was urgently transferred to our hospital because of threatened premature labor when the Kumamoto earthquakes hit the area where she lived. During her hospitalization, she complained of gradually increasing symptoms of polyuria and polydipsia. The serum level of arginine vasopressin (AVP) was 1.7 pg/mL, which is inconsistent with central DI. The challenge of diagnostic treatment using oral 1-deamino-8-D-AVP (DDAVP) successfully controlled her urine and allowed for normal delivery. DDAVP tablets were not necessary to control her polyuria thereafter. Based on these observations, clinical diagnosis of GDI was confirmed. Pathophysiological analyses revealed that vasopressinase expression was more abundant in the GDI patient’s syncytiotrophoblast in placenta compared with that in a control subject. Serum vasopressinase was also observed during gestation and disappeared soon after delivery. Vasopressinase is reportedly identical to oxytocinase or insulin regulated aminopeptidase (IRAP), which is an abundant cargo protein associated with the glucose transporter 4 (GLUT4) storage vesicle. Interestingly, the expression and subcellular localization of GLUT4 appeared to occur in a vasopressinase (IRAP)-dependent manner.

**Conclusion:**

Because placental volume may be associated with vasopressinase overproduction in GDI, vasopressinase (IRAP)/GLUT4 association appears to contribute to the growth of placenta in this case.

## Background

Diabetes insipidus (DI) during pregnancy is characterized into three categories, 1) masked central DI that has become obvious, 2) nephrogenic DI and 3) transient DI of pregnancy (gestational diabetes insipidus or GDI) due to increased production and release of vasopressinase from growing placental tissue [1]. Among these, the prevalence of GDI is approximately 1 in 30,000 pregnancies, and patients with GDI are characterized in pregnancy by polyuria and polydipsia due to placental overproduction of vasopressinase, which is expressed in placental trophoblasts and degrades arginine vasopressin (AVP) [2, 3]. Although there is an increase in endogenous AVP production during pregnancy to maintain sufficient antidiuretic activity, increased degradation of AVP by placental vasopressinase may result in GDI. Because vasopressinase is metabolized in liver, hepatic dysfunction could increase the amount of circulating vasopressinase.

We present here a case of GDI having threatened premature labor in a 16-year-old woman with intact hepatic function who was successfully treated with oral DDAVP tablets. We also performed pathophysiological investigation using serum and placental tissue from the patient.

## Case presentation

A 16-year-old Japanese primipara was urgently transported from a regional maternity clinic to our hospital because of threatened premature labor in the 25th week of pregnancy. There was no medical or family history of note. At that time, there had been extremely powerful earthquakes occurring in the Kumamoto area where she lived, and as a result, she had been forced to stay in an emergency shelter. When she arrived at our hospital, treatment with ritodrine hydrochloride, magnesium sulfate, betamethasone and hydroxyprogesterone was initiated, and her premature uterine contractions were successfully controlled.

**Fig. 1:**
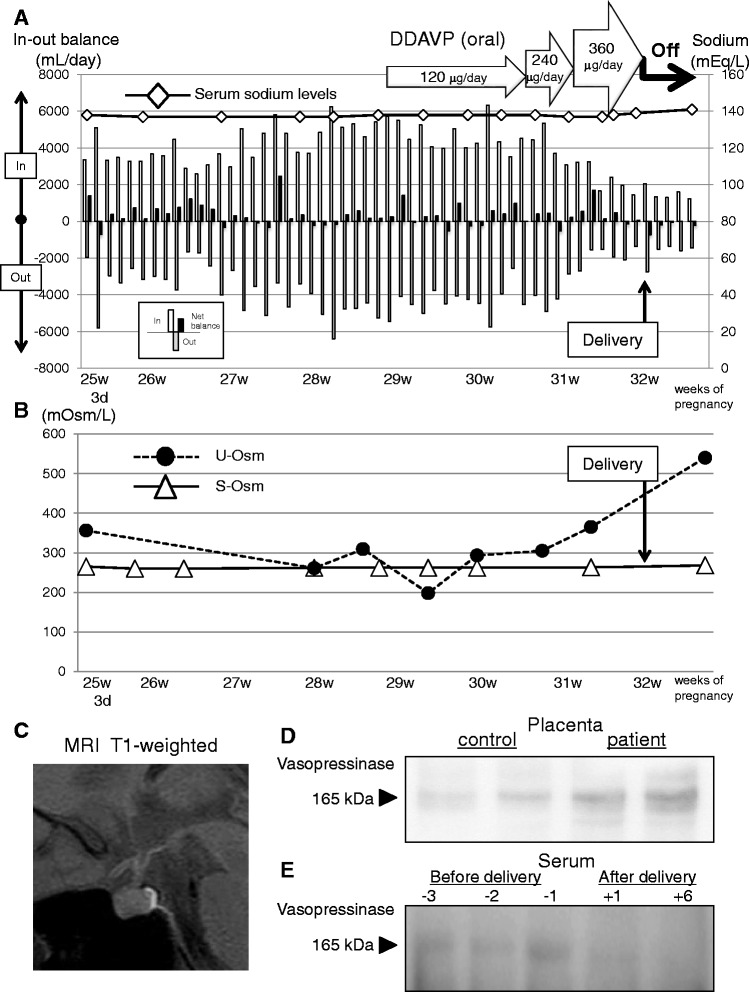
**a** Fluid intake, urinary output, serum sodium levels and DDAVP treatment in the patient’s pregnancy course. The open, gray and closed column represents fluid intake, urinary output and net water balance, respectively. **b** Temporal changes of serum and urinary osmolality in pregnancy course of the patient. **c** Transverse T1-weighted MRI imaging obtained right after delivery. **d** Protein samples were prepared using placental tissues from the patient and a gestational age-matched control subject, and were subjected to western blotting for vasopressinase expression. Placental protein samples were isolated and prepared from two independent parts from these subjects. **e** Protein samples were prepared using serum from the patient at different time points, and were subjected to western blotting for vasopressinase expression

Soon after her admission, considerable polyuria (3000–6000 mL/day), nocturia (5–6 times a night) and polydipsia gradually became obvious (Fig. 1a). Because she was restricted to bedrest to prevent premature labor, precise diagnosis and treatment of polyuria was necessary. From the 27th to the 28th week of gestation, the amount of urine increased from 4000 to 6000 mL/day (Fig. 1a). Fasting plasma glucose levels during hospitalization were around 78–88 mg/dL and the results from a 75-g oral glucose tolerance test indicated that she was not diabetic (Table. 1). Although she developed polyuria, serum sodium levels were constant at around 137–140 mEq/L during the clinical course (Table. 1a). Serum osmolality was maintained at around 260–265 mOsm/L, while urine osmolality (191–293 mOsm/L) showed below the levels of serum osmolality, indicating that her urine concentration ability had deteriorated (Fig. 1b). The serum level of AVP was 1.7 pg/mL (0.3–3.5; Table 1), which is inconsistent with central DI. At the time of admission, hepatic dysfunction was not observed (Table 1). A water deprivation test would have been unsuitable for diagnosis in this case because dehydration can deteriorate the maternal-fetus environment. Although a precise diagnosis of GDI was not made at that time, we decided to use oral 1-deamino-8-D-AVP (DDAVP) tablets for diagnostic treatment to determine whether her urine would respond to DDAVP and treat DI, as well as to rule out nephrogenic DI. Thus, the patient was first given 120 μg of oral DDAVP tablets, and then the dose of DDAVP was gradually increased to 360 μg until the amount of urine was less than 2000 mL/day (Fig. 1a) with a urine osmolality of 305–365 mOsm/L (Fig. 1b). At the 32nd week of gestation, fetal maturation was confirmed and medical support to prevent premature contractions was ceased. Soon after this decision, a male fetus weighing 1660 g and 45 cm was delivered with a one-minute Apgar score of 8/9. Immediately after the normal delivery, DDAVP tablets were no longer necessary to control her polyuria. Daily urine amount decreased to 1300–1600 mL/day (Fig. 1a). At this point, s-Osm was 268 mOsm/L and u-Osm increased to 540 mOsm/L without DDAVP treatment (Fig. 1b). Pituitary magnetic resonance imaging (MRI) was performed and confirmed that the normal bright spot in the posterior pituitary on the T1 image was present (Fig. 1c). After these observations, clinical diagnosis of GDI was confirmed.

**Table 1:** Laboratory data around admission

Complete Blood Count	
WBC	10,700 /μL
RBC	3.43 × 10^6^/μL
Hgb	10.2 g/dL
Hct	31.6%
MCV	92.1 fL
MCH	29.7 pg
MCHC	32.3%
PLT	306 × 10^3^/μL
Neutro	77.5%
Baso	0.2%
Eosino	2.3%
Lymph	15.8%
Mono	4.2%
Endocrinological exam	
ACTH	14.24 pg/mL
Cortisol	7.8 μg/dL
GH	2.7 ng/mL
IGF-1	211 ng/mL
LH	3.3 mIU/mL
FSH	2.2 mIU/mL
PRL	10.7 ng/mL
TSH	4.24 μIU/mL
F-T3	2.86 pg/mL
F-T4	1.05 ng/dL
PRA	13.0 ng/mL/h
PAC	618.0 pg/mL
s-Osm	265 mOsm/kg
ADH	1.7 pg/mL (0.3~ 3.5)
intact PTH	30 pg/mL
TRAb	9.4%
TPO Ab	5 IU/mL
Tg Ab	193.7 IU/mL (< 28.0)
Blood chemistry	
T-P	5.9 g/dL
Alb	3.3 g/dL
T-Bil	0.4 mg/dL
AST	12 U/L
ALT	9 U/L
LD	144 U/L
γ-GTP	7 U/L
LAP	81 U/L
CHE	259 U/L
ALP	288 U/L
LDL-C	75 mg/dL
HDL-C	75 mg/dL
TG	33 mg/dL
CPK	75 mg/dL
BUN	10.4 mg/dL
Crea	0.46 mg/dL
eGFR	> 90 mL/min/1.73m^2^
Na	137 mEq/L
K	3.2 mEq/L
Cl	104 mEq/L
Ca	8.1 mg/dL
IP	3.4 mg/dL
Fe	119 μg/dL
CRP	0.05 mg/dL
FPG	80 mg/dL
HbA1c	4.9%
Blood glucose at GTT 60 min: 118 mg/dL, 120 min: 135 mg/dL	
Urine data	
U-Osm	261 mOsm/kg
U-Na	37 mEq/L
U-K	12 mEq/L

*PRA* Plasma renin activity, *PAC* Plasma aldosterone concentration, *TPO Ab* Thyroperoxidase antibody, *Tg Ab* Thyrpoglobulin antibody

At 2 weeks *post-partum*, the patient’s sodium, serum and urine osmolality were within normal range. She remained clinically well, and is currently breast-feeding with no complications.

At 4 weeks *post-partum*, she developed postpartum destructive thyrotoxicosis with increases of free-T4 and free-T3, and suppressed thyroid stimulating hormone (TSH), with positive thyroglobulin antibody (Table 1). After 8–12 weeks of follow up with no medications, she had completely recovered thyroid function.

## Pathophysiological investigations

### Subjects and methods

#### Informed consent

Written informed consent was obtained from the patient and her mother, and a gestational age matched that of normal control subject. This investigation was approved by the institutional review board at Kumamoto University (Ethics No. 1192).

#### Western blotting

Plasma AVP concentrations were measured using a double-antibody radio-immuno assay (AVP RIA Neo LSI-M kit; LSI Medience Corporation, Tokyo, Japan). Lysate isolated from placental tissues or serum samples was subjected to SDS-PAGE followed by western blotting. The blots were probed with the vasopressinase antibody (Cell Signaling, Danvers, MA: cs-6918), glucose transporter 4 (GLUT4; Santa Cruz, Dallas, TX: sc-53,566), Akt (Cell Signaling: cs-9271) or phospho-Akt (Cell Signaling: cs-9272) and HRP-conjugated secondary antibody obtained from Santa Cruz. Membrane and cytosolic fraction were isolated using ProteoExtract Transmembrane Protein Extraction Kit (EMD Millipore, Darmstadt, Germany).

#### Immunohistochemistry

To identify the vasopressinase or GLUT4 expression and localization in placenta, immunohistochemical staining using vasopressinase or GLUT4 antibody was performed. Frozen thin sliced placental samples 10 μm in thickness from the patient and a gestational age-matched control subject were prepared. Incubation with these antibodies (1/100 dilution) was performed overnight at 4 °C. After washing, the sections were incubated with secondary antibody conjugated with Alexa Fluor 488 or 555 (Molecular Probes Inc., Eugene, OR) for 1 h at room temperature. Nuclei were stained with DAPI (1/200 dilution) at room temperature for 5 min. After rinsing, the sections were mounted with Fluoromount mounting medium (Diagnostic BioSystems, Pleasanton, CA) and examined with a fluorescence microscope (BZ-9000; Keyence, Osaka, Japan).

The appearance of her placental tissue was normal and consistent with normal placentas as described in previous report [4]. The weight of the placenta was 425 g at 32 weeks. At this age, mean placental weight is 325 ± 77 g (lower 10%: 241 g and upper 90%: 436 g), indicating that the weight of placenta was relatively heavy [5]. Placental weight and birth weight ratio for a male infant in this case was 0.256, which is approximately the 50th percentile of the normal distribution of placental/birth weight ratio [6].

Then, we performed western blotting of the serum and placenta, and immunohistochemical analysis using placental tissue from the patient and a gestational age-matched normal control subject. Placental expression of vasopressinase in this GDI patient was approximately 5 times more abundant compared with that in the control subject (Fig. 1d). The presence of vasopressinase in the patient was apparent in sera taken 3, 2 and 1 days prior the delivery, but the serum levels of vasopressinase became undetectable immediately after delivery (Fig. 1e).

**Fig. 2:**
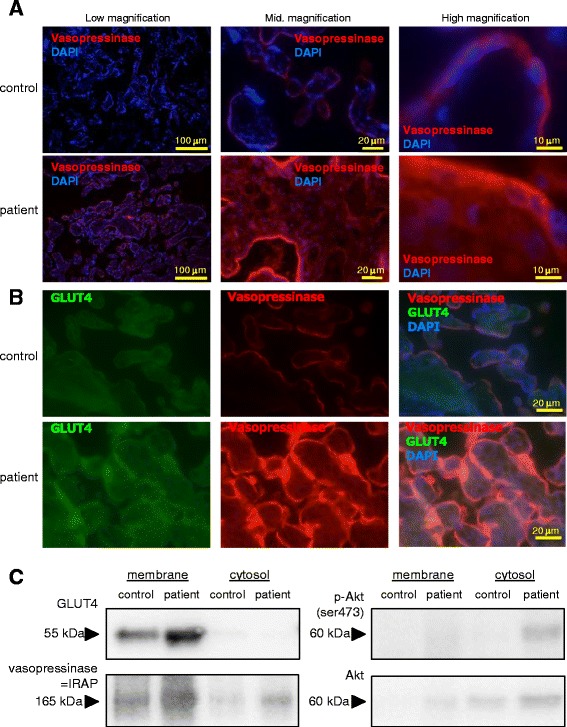
Frozen sections of placental tissues from the patient and a gestational age-matched control subject were fluorescent-immunohistochemically stained for vasopressinase (**a**) or vasopressinase and GLUT4 (**b**) with DAPI. **c**: Membrane or cytosolic fractions of samples were extracted from the placental tissues of the patient and the gestational age-matched control subject, and subjected to western blotting for GLUT4, vasopressinase (identical to IRAP), Akt or phosphor-Akt (ser473)

Placental vasopressinase expression was also confirmed by fluorescent immunohistochemistry. This analysis showed that the pattern of vasopressinase expression was restricted, and more abundant in GDI syncytiotrophoblast compared with that in the control placenta (Fig. 2a). It should be noted that vasopressinase was also expressed in normal placental syncytiotrophoblast to some extent (Figs. 1d and 2a).

Vasopressinase is also known as insulin-regulated aminopeptidase (IRAP), an abundant cargo protein associated with GLUT4 storage vesicle (GSV). Double staining of GLUT4 with vasopressinase (IRAP) revealed that these two proteins were co-localized in the syncytiotrophoblast of the GDI placenta (Fig. 2b). The expression of GLUT4 was also limited in the syncytiotrophoblast of the GDI placenta, and the amount of GLUT4 and vasopressinase (IRAP) was increased in the GDI placenta (Fig. 2b). Subcellular localization analysis indicated that both vasopressinase (IRAP) and GLUT4 were observed predominantly in membrane fraction, but much less in cytosol (Fig. 2c). The levels of vasopressinase (IRAP) and GLUT4 were both greater in GDI placenta than those in the control (Fig. 2c). As GLUT4 membrane translocation is predominantly dependent on insulin signaling, we examined Akt activation in placental tissues. Phosphorylation of Akt in both the control subject and the patient appeared to be negligible.

## Discussion

GDI is a rare complication during pregnancy with an estimated prevalence of 1 in 30,000 pregnancies [2, 3]. This form of DI is slightly more common in women with multiple gestations, subclinical or masked central DI, or liver dysfunction because of the impairment of the degradation of vasopressinase. It usually becomes symptomatic at the end of the 2nd or 3rd trimester of pregnancy and remits spontaneously days to a few weeks after delivery [1]. The fact that this case clinically resolved soon after delivery is supported by the results showing immediate disappearance of vasopressinase in serum examined by western blot analysis. In most GDI cases, polyuria is resolved days to weeks after delivery [1]. Although the reason for immediate clearance of serum vasopressinase is not elucidated, degradation of vasopressinase in the liver might be augmented in this case.

Because central DI or nephrogenic DI can also be observed during pregnancy, precise differential diagnosis and prompt treatment is important. Serum AVP level showed within the normal range, indicating that central DI could be ruled out. Although water deprivation test was clinically risky in this case, MRI imaging during pregnancy can be safely performed, and may provide us further confirmation that this was not a case of central DI. Even though clinical diagnosis of GDI was not confirmed at that time, use of DDAVP as diagnostic treatment was implemented to control her urine as well as to rule out nephrogenic DI. Final diagnosis of GDI was determined by DDAVP responsiveness and immediate resolution of polyuria after delivery. From the clinical point of view, hypotonic (191–293 < 300 mOsm/L) polyuria (3000–6000 mL) was confirmed, but serum osmolality remained low around 260–268 mOsm/L, with plasma sodium levels around 137–140 mEq/L, both of which are inconsistent with DI. As shown in Fig. 1a, her fluid intake almost always exceeded the amount of urine; thus she may not have developed dehydration and hemoconcentration.

The mechanism of GDI development is considered to comprise an increase in the amount of placental vasopressinase, which degrades AVP in circulation, which finally results in impairment of urine concentrating ability. Vasopressinase is expressed mainly in the placental syncytiotrophoblast during pregnancy and the amount is correlated with the volume of the placenta. Indeed, the placental weight was slightly higher (425 g: approximately 90th percentile at 32 w of gestation [5]) whereas the placental/birth weight ratio was normal in this case. Expression of vasopressinase is regulated by transcription factors such as activator protein-2 (AP-2), selective promoter factor 1 and nuclear factor-1. These relevant cis-acting elements are located in the 5′-region in the vasopressinase genomic sequence. Among these, AP-2 may be the most important transcription factor regulating vasopressinase gene expression in human placenta [7]. Although the gene regulation of vasopressinase is not elucidated in this case, the overexpression of vasopressinase in placenta could be associated with GDI.

Vasopressinase is also known as oxytocinase, placental leucine aminopeptidase, leucyl/cystinyl aminopeptidase or insulin-regulated aminopeptidase (IRAP), and works as a zinc-dependent aminopeptidase that cleaves vasopressin as well as oxytocin, bradykinin, enkephalin, dynorphin A or other small peptide hormones. Interestingly, vasopressinase (IRAP) is localized in GLUT4 that contains GSV of insulin-sensitive tissues such as muscle and fat [8]. GLUT4 is also reportedly expressed in the placental syncytiotrophoblast in the early stage of gestation and may mediate glucose uptake for rapid growth of the placental tissues [9]. However, it is not known whether GLUT4 is co-expressed with vasopressinase (IRAP) in the syncytiotrophoblast in patients with GDI in the later stage of gestation. We first demonstrated the co-expression of vasopressinase (IRAP) with GLUT4 by immunohistochemical staining in the placental syncytiotrophoblast in this case. Surprisingly, both vasopressinase (IRAP) and GLUT4 are almost exclusively expressed in the membrane compartment and may play a role in enhancing the growth of the placenta.

The amount of GLUT4 is up-regulated in GDI placenta (mainly in membrane fraction) compared with that in normal control subject, indicating that vasopressinase (IRAP) may control GLUT4 not only in GSV membrane translocation but also in protein abundance. Indeed, GLUT4 levels are diminished by 40–85% in global IRAP knockout mice [10], exhibiting normal reproduction and maternal behavior [10, 11]. Amino terminus of IRAP overexpression causes GLUT4 membrane translocation [12]. Our observation suggests that, in the pathophysiological situation of GDI, vasopressinase (IRAP) regulates the amount and subcellular localization of GLUT4 in the later stage of gestational placenta. This hypothesis has to be evaluated and confirmed using more GDI cases with pathophysiological examinations.

We hypothesize that the Kumamoto earthquakes may have played a part in triggering this patient’s premature contractions. Indeed, earthquake exposure has been shown to result in a significant decline in gestational age and increase in preterm delivery [13]. As stress-induced oxytocin has uterus-contracting properties [14], oxytocinase can be produced from the placenta to prevent premature labor. Because oxytocinase is identical to vasopressinase, circulating vasopressin was degraded, resulting in GDI, and GDI is associate with premature birth [15]. Because placental volume may be associated with vasopressinase overproduction in GDI and vasopressinase (IRAP) may positively regulate GLUT4 expression and membrane translocation, this IRAP/GLUT4 association could contribute to the growth of placenta, thus the symptoms of GDI may be augmented later on.

Postpartum destructive thyrotoxicosis, painless thyroiditis or silent thyroiditis is relatively common in women in the postpartum period. Despite an extensive search of the literature, no case of GDI followed by or co-existing with postpartum destructive thyrotoxicosis could be found. As a result of autoimmune mechanisms, postpartum destructive thyrotoxicosis can be observed during the clinical course of chronic thyroiditis (Hashimoto thyroiditis) [16]. Central DI associated with lymphocytic hypophysitis may be associated with chronic thyroiditis [17], but was not in this case. Because there are a variety of etiologies underlying the mechanism of GDI, GDI followed by postpartum destructive thyrotoxicosis may occur. However, it is important to monitor the endocrine function and symptoms in the pregnant mother during and after the pregnancy.

## Conclusions

Vasopressinase is strongly expressed in the placental syncytiotrophoblast, and is released into serum to degrade AVP, which results in GDI. Vasopressinase (IRAP) overproduction may enhance GLUT4 expression and membrane translocation, contributing to the growth of placenta during the later stage of gestation. This placental vasopressinase (IRAP)/GLUT4 association in this GDI case may regulate multiple aspects such as uterus contraction, water balance and placental growth.

## Abbreviations

DDAVP: 1-deamino-8-D- arginine vasopressin
GDI: gestational diabetes insipidus
GLUT4: glucose transporter 4
IRAP: insulin regulated aminopeptidase
